# Array of Resonant Electromechanical Nanosystems: A Technological Breakthrough for Uncooled Infrared Imaging

**DOI:** 10.3390/mi9080401

**Published:** 2018-08-14

**Authors:** Laurent Duraffourg, Ludovic Laurent, Jean-Sébastien Moulet, Julien Arcamone, Jean-Jacques Yon

**Affiliations:** 1 Université Grenoble Alpes, F-38000 Grenoble, France; ludovic.laurent@mirsense.com (L.L.); jean-sebastien.moulet@cea.fr (J.-S.M.); julien.arcamone@cea.fr (J.A.); jean-jacques.yon@cea.fr (J.-J.Y.); 2 CEA, LETI, Minatec Campus, F-38054 Grenoble, France

**Keywords:** nano resonator, nano-system array, uncooled IR-bolometer

## Abstract

Microbolometers arethe most common uncooled infrared techniques that allow 50 mK-temperature resolution to be achieved on-scene. However, this approach struggles with both self-heating, which is inherent to the resistive readout principle, and 1/f noise. We present an alternative approach that consists of using micro/nanoresonators vibrating according to a torsional mode, and whose resonant frequency changes with the incident IR-radiation. Dense arrays of such electromechanical structures were fabricated with a 12 µm pitch at low temperature, allowing their integration on complementary metal-oxide-semiconductor (CMOS) circuits according to a post-processing method. H-shape pixels with 9 µm-long nanorods and a cross-section of 250 nm × 30 nm were fabricated to provide large thermal responses, whose experimental measurements reached up to 1024 Hz/nW. These electromechanical resonators featured a noise equivalent power of 140 pW for a response time of less than 1 ms. To our knowledge, these performances are unrivaled with such small dimensions. We also showed that a temperature sensitivity of 20 mK within a 100 ms integration time is conceivable at a 12 µm pitch by co-integrating the resonators with their readout electronics, and suggesting a new readout scheme. This sensitivity could be reached short-term by depositing on top of the nanorods a vanadium oxide layer that had a phase-transition that could possibly enhance the thermal response by one order of magnitude.

## 1. Introduction

Microelectromechanical systems (MEMS) are either used according to a static mode (accelerometers, micro-mirrors, radio frequency (RF) switches, bimorph structures) or rather in a dynamic way, when the harmonic response of the system is requested. Although quartz crystals were first studied (and continue to be widely used as time references), silicon is most likely the most widely used material for MEMS and NEMS (nano-electromechanical systems) due to its excellent mechanical properties. Indeed, advances in microelectronics on silicon wafers (increasingly large diameters—the reliability and reproducibility of manufacturing methods, silicon on insulator (SOI) substrates) have strongly enabled the rise of silicon-based MEMS/NEMS. Beyond silicon, alternative materials are nowadays already industrialized, such as lead zirconate titanate (PZT) and AlN, or envisaged such as GaAs, graphene, or aluminum, generally deposited on silicon substrates (in particular for economic reasons). Finally, the intrinsic compatibility of the manufacturing of silicon or silicon-based materials with a complementary metal-oxide-semiconductor (CMOS) integration (“above-integrated circuit (IC)” approach or co-integration approach) is an undeniable asset in order to realize a high-signal-to-noise ratio (SNR) system including an actuator/detector and a compact readout circuit that is affordable and energy efficient.

The small size of NEMS makes them particularly sensitive to their external environments, while keeping very good frequency stability. In particular, silicon-based nanoresonators have already demonstrated their formidable potential in their number of application domains. Nanoresonators are excellent physical sensors, especially for measuring forces [1,2] or mass [3,4], for gas detection [5,6], but also for measuring the temperature [7,8,9,10]. The ultimate sensitivity of the order of the yoctogram (10^−24^ g—mass of a proton) has even been demonstrated with single-wall carbon nanotubes (CNT) [11,12]. More generally, nanoresonators can extend proteomics to high mass biomolecules (such as complexes of proteins or viruses) [3,13]. There is also intense activity in the NEMS community related to the study of oscillators in their fundamental quantum mode using the reciprocal interaction of an optical micro cavity with a mechanical resonator [14,15,16]. Examples of mechanical resonator applications are plethoric: particle counting in a fluid medium [17,18], magnetometry [19,20,21,22,23], actuators [24], RF filters [25], and in biology [26,27].

**Figure 1:**
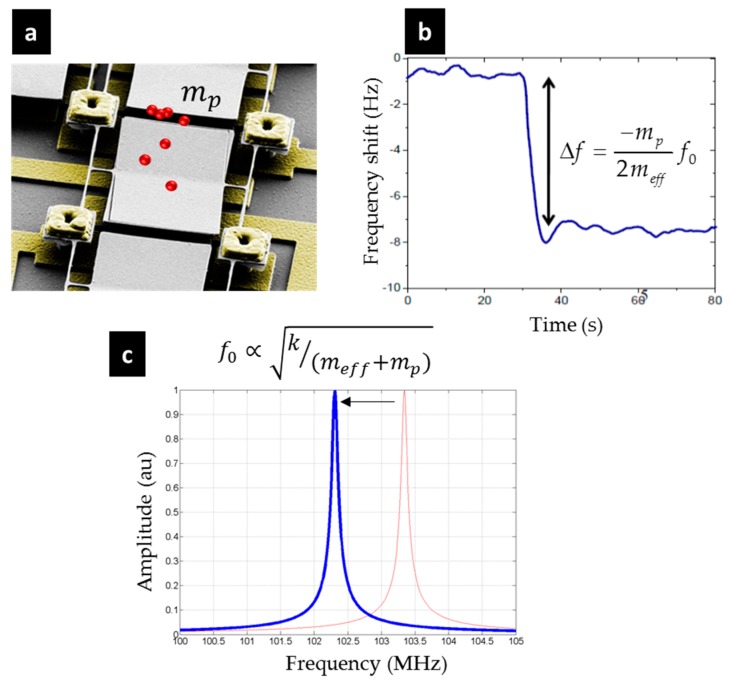
The principle of mass measurement (adapted from [28]). (**a**) Example of a nanoresonator on which particles have landed; (**b**) Shift in the frequency caused by the arrival of particles. Monitoring in real-time the resonance frequency allows us to deduce the amount of accreted mass; (**c**) From the spectral perspective: a shift in the spectrum toward low frequencies.

The measurement principle is quite simple: it consists of monitoring the frequency shift of a NEMS kept at a given vibration (at a fixed controlled amplitude) using a closed loop circuit via a “phase-locked loop” (PLL) or a self-oscillating circuit. Any change in an external physical parameter (temperature, pressure, force, acceleration etc.) or on the surface of the material (adsorption of a gas, molecule, etc.) will modify its stiffness or its mass, thus inducing a change in the resonant frequency, which is continuously measured. Figure 1, adapted from [28], illustrates the measurement principle for when a frequency shift is caused by a mass landing on top of a nanocantilever. In this case, a piezoresistive transduction was used to measure the mechanical oscillations of the cantilever (the actuation being purely capacitive [29]). Indeed, it was shown that piezoresistive detection is highly suitable at high frequency compared to the capacitive readout in a self-oscillation loop [30].

Neutral mass spectroscopy with such a system is now ongoing, and some papers have already demonstrated their interest for biomolecule analysis [3]. Beyond the ultra-sensitivity of NEMS, the overall analysis time has to be fast enough to make this technique a realistic solution. To tackle this key issue, the basic method consists of using a NEMS array for increasing the capture cross-section, and hence speeding up the analysis. In a recently published work, a NEMS array dedicated to mass sensing was realized with a frequency address, with each NEMS having a slightly different resonance frequency that labelled its position inside the array [31].

Nanoresonators can also be used for thermal sensing. Resonant NEMS arrays with a suitable driving electronics could be a way to continue to decrease the pixel pitch of the thermal imager, keeping the performance constant. Among the various current uncooled infrared focal plane array (IRFPA) technologies, the microbolometer is the most common uncooled infrared device. It operates by converting the heating of a thin suspended membrane due to IR absorption into a variation of the electrical resistance of a layer deposited on it. This layer is commonly made of a thin film of semiconductor, using mostly vanadium oxide (VOX) or amorphous silicon (a-Si), because of their high thermal sensitivity (1/*R* × d*R*/d*T*), which is about 2–3%/K. To reach a high thermal sensitivity, the membrane has to be insulated from the substrate, and it is suspended above the readout integrated circuit (ROIC) by thin and long insulation legs. Thermal insulation as high as 2 mK/W has, for instance, been reported for microbolometers with 12 µm pixel pitch [32]. Such a development has achieved noise equivalent temperature differences (NETD) at a very low level, around 50 mK (F/1 lens, 30 Hz frame rate, 300 K background) [33,34]. At ultra-small pixel pitches (less than 12 µm), the performances could be kept to the cost of a very high thermal insulator, leading to a dramatic temperature increase of the suspended plate, which can be deleterious for the electrical properties of the sensing material [35]. Both VOX and a-Si microbolometers can be affected [36]. The direct consequence is a persistent afterimage when the scene is removing [37]. This is particularly true for when the microbolometer is exposed to a high temperature source, such as fire, explosion, or the sun, and is this sometime referred to as the “Sun Burn” effect [38]. Although a shutter-based non-uniformity correction can mitigate this effect, latent images reappear rapidly after shutter operation and persist with a very long decay time. Thus, there is a clear need for a new transduction method that can withstand high temperature exposure. In parallel, this technique has to be compatible with very large scale manufacturing for future consumer markets that require small pixel-pitch and high resolution.

In this context, we suggest a new transduction method based on high-frequency mechanical nanoresonators designed to be ultra-sensitive to IR radiation. For mid-term vision, this approach intends to replace the current thermistor-based bolometers. The frequency stability of these nanoresonators should allow for the fundamental phonon noise to be reached, and to better cope with the thermal issues, while keeping a high resolution on the scene for the small pixel sizes. In principle, the excellent frequency sensitivity of nano/micro resonators makes them perfect ultra-small thermal sensors. However, two main questions must be raised: how can an efficient electrical transduction with a tiny mechanical displacement be realized without any self-heating? Is the frame rate fast enough with such an approach to obtain a net image? This paper will provide some key insights, using a CMOS-based approach.

In a first level of answer, the transduction technique has to be properly chosen. Unlike the conclusions made in previous papers [30], capacitive transduction is preferred for removing the self-heating that results in a background signal and additional noise. Ultra-small capacitance variation is however, more delicate to read out [39], and a specific buffer circuit has to be developed, as we will show later on in the paper. Many types of transductions have been suggested over the last 10 years. A second level of the answer lies in the use of the NEMS array collectively or individually actuated, and detected by a CMOS circuit that could be placed in its close vicinity. The CMOS circuit will actuate the mechanical systems at their resonance frequencies, and perform the addressing of a single NEMS or a sub-array of NEMS to be addressed. The spatial proximity of the CMOS with the thermal sensors drastically limits the effects of electrical parasitic coupling and attenuation. Limiting these effects consequently maximizes at circuit input, both the absolute value of the useful signal, and its signal-to-background ratio (SBR), resulting in maximization of the SNR at the circuit output. To conclude, this juxtaposition has significant advantages, namely the compactness of the system, and the unparalleled electrical transduction efficiency.

## 2. Design and Fabrication of Electromechanical Resonator Arrays

The basic geometry of the resonator was a suspended plate that experienced a torsional vibration around a rotation axis. Several metallic electrodes were structured underneath for electrostatic actuation and capacitive measurement of the paddle displacement. These electrodes and the paddle formed a λ/4-resonant cavity centered at 8 µm for enhancing the absorption of the incident IR-radiation. The Fabrication process and the materials had to be temperature-compatible with monolithic integration, in order to simplify the manufacturing of the imagers and their integration on a CMOS readout circuit. Thus, the materials must meet three main criteria: (i) good mechanical features; (ii) low thermal conductivity; (iii) low-temperature deposition. The critical dimensions (as the width legs ensuring the rotation and the thermal insulation of the plate) had to be well controlled during the fabrication process to ensure that all pixels were functional inside the imager.

**Figure 2:**
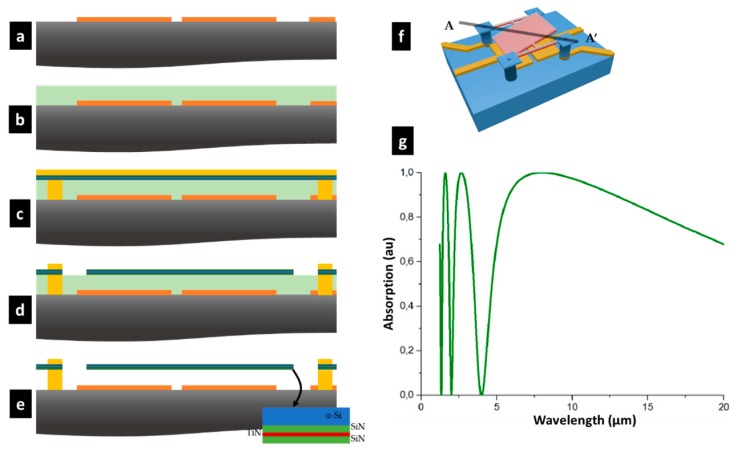
Synopsis of the fabrication process along the cross section AA’: (**a**) Deposition of a 300 nm thick AlCu layer on a silicon substrate/strip lines/reflector wet etching; (**b**) 2 µm deposition of a polyimide layer; (**c**) Etching of the polyimide layer to build up the metal studs, and deposition of the plate material; (**d**) Definition of the plate; (**e**) Dry etching O_2_ plasma release; (**f**) 3D artist view of a pixel; (**g**) Absorption spectrum of the 2 µm thick micro cavity.

The low-temperature fabrication process was derived from classical bolometers (i.e., a deposition process <300 °C and above-IC compatible). First, a 300 nm thick AlCu layer was deposited on a silicon substrate and structured to form the transduction electrodes. A 2 µm-deposition of a polyimide layer was then formed and constituted the sacrificial layer. The latter was opened to build up the metal studs that would insure the mechanical support and the electrical connection with the electrical connections below. Two silicon nitride (SiN) layers of 10 nm encapsulated a titanium nitride (TiN) layer that acted as an electrode as well as an absorber. The TiN thickness was defined to be impedance-matched with the vacuum (*Z*_0_ ~ 376 Ω), in order to obtain a direct absorption rate that was close to 50%. The λ/4-optical cavity (2 µm thick) between the aluminum–copper electrodes and the TiN layer allowed an absorption efficiency of 80% to be reached over the 8–14 µm wavelength range. Figure 2g shows the spectral absorption of such a cavity with this specific SiN/TiN/SiN/a-Si stack in this wavelength range.

The encapsulation of the TiN layer was performed for stress compensation reasons and to protect it during the release step. An amorphous silicon of 150 nm thickness (a-Si) was deposited on the top SiN to stiffen the plate. The plate had to be polarized through the legs via the thin TiN layer. Electrostatic actuation was possible in these conditions, since no high current was required. Electrical contacts were made by opening the top layers (SiN and a-Si).

**Figure 3:**
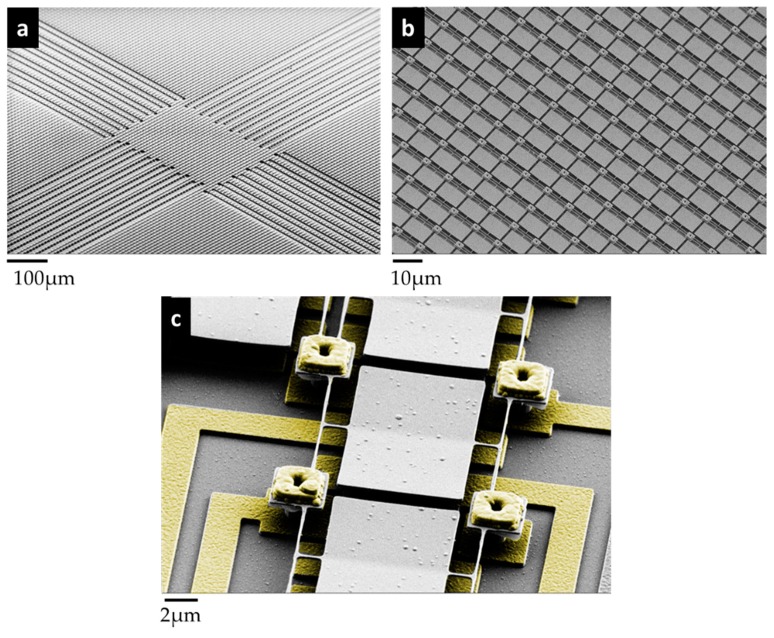
Scanning electron microscopy (SEM) pictures of an array of electromechanical pixels fabricated with a low-temperature process: (**a**) Large field view of an array; only the central 96 × 96 array is connected to electrical pads; pixels above the connection wires have been removed to avoid any cross-talk; (**b**) Zoom-in on the center of the array; (**c**) SEM picture of a typical H-shape pixel; Nanorod length = 1.5 µm, width = 250 nm and thickness = 180 nm (insulation arm length = 8.6 µm).

**Figure 4:**
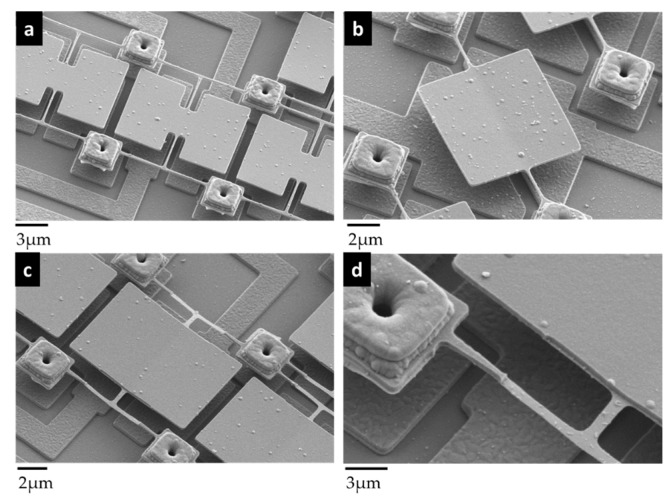
SEM pictures of alternative versions derived from the nominal design: (**a**) Butterfly-shaped pixel with longer rods; (**b**) Simple pixel without insulating legs; (**c**) H-shape pixel with thinner nanorods for enhancing the thermal insulation; rod-thickness = 30 nm; (**d**) Zoom of the legs (Figure 4c) attached to a stud that acts as a mechanical anchor and that provides electrical contact with the lines underneath.

The torsional mechanical eigenmode has always been addressed in all designs of pixels so far. Indeed, the advantages of the torsional mode are threefold: (i) this mode is less sensitive to the residual axial stress that could be different from one side to the other side of an array [9,40]; (ii) the dynamic range set by the onset of nonlinearity is higher for the torsional mode compared to the flexural modes [9,40,41,42], since only the external fiber of the rods experiences a strain [43]; (iii) the paddle surface remains large compared to the overall resonant body and makes a capacitive actuation easy. Resonator arrays of 666 × 520 pixels, with a 12 µm-pitch were fabricated. Figure 3a shows a scanning electron microscopy (SEM) picture of a typical array. The first electromechanical tests were achieved using polarization lines structured below the pixel. This interconnection can be observed on Figure 3a, and enabled the actuation and read out of an array of 96 × 96 electromechanical pixels. An SEM zoom-in of a typical H-shape pixel is presented in Figure 3b,c. The nano-rod length was 1.5 µm for a cross-section of 250 nm × 180 nm (width × thickness). The insulation arm length was 8.6 µm. This design was the nominal version of our electromechanical pixel. Other versions were, however, realized and some of them are presented in Figure 4a–d. These alternative versions will be reviewed in the next section: they were conceived in an attempt to meet the best trade-off between an efficient thermal insulation and a large mechanical dynamic range, these two key features being antagonists. Indeed, it is interesting to look at the Equation (1) below. It shows that the thermal response is inversely proportional to the thermal conductance, *G*. The thermal insulation should be improved by increasing the lengths of the legs and the rods. At the same time, the torsional stiffness should be high enough to prevent the occurrence of quick and strong nonlinear effects. This requires quite short legs. The lengths and widths of the legs versus the rods had to be carefully chosen to find the best operating point (large linear displacement, large thermal insulation, and low driving voltages). Notice that even if the asymmetric designs were drawn to enhance the electrostatic actuation, a square shape could be realized. Along the two directions, the pitch was nevertheless kept constant at 12 µm.

**Figure 5:**
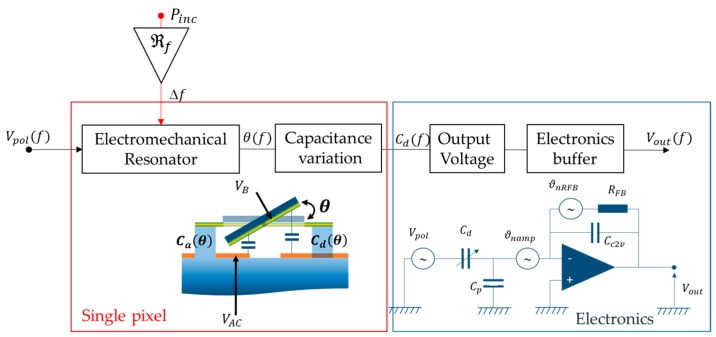
Synopsis of the open-loop measurement chain: The red box corresponds to a single electromechanical pixel that translates the incident IR-radiation *P_inc_* on the scene into a resonance frequency shift; the blue box corresponds to the close-by electronics that convert the mechanical oscillations into an electrical signal; *V_pol_* is the polarization of the pixel; *θ*(*f*) is the angular oscillation of the paddle around the rods; *Cd*(*f*) is the induced capacitance variation used to read out the signal; *V_out_*(*f*) is the output signal supplied by the buffer. *C_p_* is the total capacitance due to amplifier input capacitance, and parasitic capacitances between the electrical connections and the ground.

For the sake of clarity, a short introduction to the key mechanisms and noise sources is presented below. We did not aim at detailing the thermo-electromechanical equations that described the overall interactions between the mechanics and the IR-light. Rather, we gave key expressions for catching up this approach that may constitute a new paradigm in the field of IR-imaging. The overall measurement system, including a single pixel, is depicted in Figure 5. The expressions of parameters shown in this figure are detailed step-by-step below for a comprehensive vision. The further expressions are appropriate under a small-displacement (small deflection angle) assumption. The polarization of the pixel is set to keep the angular vibration in its linear range at the chosen torsional resonance frequency. *V_B_* is the bias voltage applied on the paddle (through the studs), and *V_AC_* is the sinusoidal polarization applied on the actuation electrode through the capacitance Ca. This signal can be applied with an external RF-source, in particular for the first electromechanical characterizations, but can come from the feedback loop in the case of a closed loop. The actuation frequency *f* is swept to measure the electromechanical response and the resonance frequency *f*_0_.

Basically, the electromechanical pixel converts the incident IR-optical power *P_inc_* into a resonance frequency shift ∆*f* according to a sensitivity *Rf*, which depends on both the thermal conductance of the paddle insulation (through insulation legs between the torsional rods and the plate) and the temperature coefficient of frequency:

(1) $$\Delta f=\frac{\alpha_{T}f_{0}\beta \eta }{G\left|1+j2\pi \nu \tau_{th}\right|}\;P_{inc}=f_{0}R_{f}P_{inc}$$

where *τ_th_* = *C*/*G* is the thermal time constant of the sensor, $C={\left(\frac{\partial U}{\partial T}\right)}_{V}$, the thermal capacitance at constant volume, G is the thermal conductance, $\alpha_{T}$ is the temperature coefficient of frequency (TCF) (typically −60 ppm/°C for silicon), *β* is the pixel fill factor, *η* is the bolometer absorption, *f*_0_ is the resonance frequency, and *v* the frame rate of the electronic readout. The thermal conductance is mainly due to the thermal conductance of heat through the legs. The other sources of thermal leaks—radiative and heat conductance through air—are negligible.

The capacitance variations can be calculated from geometrical considerations. After cumbersome mathematical manipulation, the final expressions can be approximated as:

(2) $$C_{a}\left(\theta \right)\approx -C_{0}\frac{\theta_{max}}{\theta }\ln\left(1-\frac{\theta }{\theta_{max}}\right)$$

(3) $$C_{d}\left(\theta \right)\approx C_{0}\frac{\theta_{max}}{\theta }\ln\left(1+\frac{\theta }{\theta_{max}}\right)$$

$C_{0}\;=\;\varepsilon_{0}\frac{L_{p}W_{p}/2}{g}$ and $\sin\left(\theta_{max}\right)\;=\;\frac{g}{W_{p}/2}$. *C*_0_ and *θ_max_* are respectively the capacitance value at rest, and the maximum deflection angle. The deflection angle is directly computed from the dynamic equation:

(4) $$J\ddot{\theta }+b\dot{\theta \;}+\kappa \theta =T_{e}\Delta f=\frac{\alpha_{T}f_{0}\beta \eta }{G\left|1+j2{\pi \nu \tau }_{\mathrm{th}}\right|}P_{\mathrm{inc}}=f_{0}R_{f}P_{\mathrm{inc}}\;T_{e}=\frac{1}{2}\frac{dC_{a}}{d\theta }V_{pol\;}^{2}+\;\frac{1}{2}\frac{dC_{d}}{d\theta }V_{B}^{2}\;J=\frac{M_{p}W_{p}^{2}}{12};\;\kappa =\frac{2GI_{r}}{L_{r}}\;\mathrm{and}\;G=\frac{E}{2\left(1+\nu \right)}$$

*T_e_* is the electrostatic torque. *J* is the moment of inertia of the paddle, assuming that the inertia moment of rods is negligible. *k*, *G*, and *I_r_* are respectively the rod torsional stiffness, the shear modulus, and the torsional quadratic moment of the rectangular suspended rods ($I_{r}=w_{r}t_{r}^{3}\left(\frac{1}{3}\;-\;0.21\frac{t_{r}}{w_{r}}\left(1\;-\;\frac{1}{12}\frac{t_{r}^{4}}{w_{r}^{4}}\right)\right),\;w_{r}\;>\;t_{r}$.). *E* and *v* are the equivalent Young modulus and Poisson ratio of the stack. 

Under the assumption of a linear regime (and small deflection amplitude of the paddle), the angle can be rewritten in the Fourier space:

(5) $$\theta \left(f\right)=\frac{T_{e}}{J}\frac{1}{f_{0}^{2}\;-\;f^{2}\;+\;j\frac{ff_{0}}{Q}}$$

Table 1 presents the values of the main features of Equations (1)–(5) for our typical electromechanical pixel. Some parameters are compared with data from literature.

At this stage, we have to struggle with a strong signal attenuation due to a capacitive bridge formed by parasitic capacitances from the metallic pads, connections and the input impedance of the final readout electronics board: VBΔC(C0 + Cp). The order of magnitude of an expected capacitance variation ΔC is around 10 aF for a $C_{0}\sim 200\;\mathrm{aF}$ and $C_{p}\sim 10\;\mathrm{pF}$. In this condition, the output signal is divided by a factor ~10^6^. This attenuation of the signal can be deleterious for obtaining a high-enough SBR to initiate a self-oscillation within a closed-loop. A way to address this issue can be through the use of a semitone-actuation (at f02): $V_{pol}\;=\;V_{AC}\cos\left(\frac{2\pi ft}{2}\right)\;-\;V_{B}$. In this case, the electrostatic torque is proportional to $V_{AC}^{2}/2$, which reduces the coupling between the actuation signal and the output signal. A differential measurement can also be added to further improve the SBR. In this scheme, two identical pixels are used to cancel out the common modes. A more complex approach based on the down-mixing method [45] can be used to get rid of the parasitic capacitances. In particular, the bias voltage is no more constant and is modulated: $V_{B}\;=\;V_{B0}\cos\left(2\pi ft\;+\;\Delta f\right)$ where Δf ≪ f. A comparison between different readout modes is shown in Table 2. We notice a quite strong improvement of the *SBR*. However, in the best cases, the signal-to noise-ratio (SNR) was lower than 20 dB, which did not guarantee a functional closed-loop.

In conclusion, to reach higher SBR (40 dB can be considered as a suitable value for the PLL) a dedicated off-chip buffer was developed to cope with the tiny capacitance variation and it was placed in the close vicinity of the pixels under test. Its schematic is presented in Figure 5. The capacitance variation is read through an intermediate circuit that measures the charge carrier variation, the applied voltage being kept constant. The current is read with a feedback capacitance, $C_{C2V}$, instead of a resistor to minimize the background, as shown in Figure 5. Thus, the output voltage is proportional to this feedback capacitance as Vout=VpolδC(θ(f))CC2V, where $\delta C\left(\theta_{r}\right)$ is the capacitance variation resulting from the motion of the paddle. $C_{C2V}$ must be chosen to be as low as possible to maximize the output signal but should be high enough to avoid unwanted effects due to parasitic capacitances (and $k_{B}T/C$ noise) as shown in the Equation (6). A 1 pF feedback capacitance in parallel with a 10 GΩ resistor were set to prevent any saturation of the output signal caused by the direct current (DC).$\;C_{d}$ and $C_{p}$ are respectively the sensor capacitance and the total input capacitance of the amplifier, respectively. This electrical scheme corresponds to a first-order filter [44]:

(6) $$V_{out}=V_{pol}\frac{C_{d}}{C_{C2V}}\left|\frac{1}{\left(1-j\frac{f_{c}}{f}\right)\left(1+j\frac{f}{f_{CO}\frac{C_{C2V}}{C_{p}}}\right)}\right|$$

**Figure 6:**
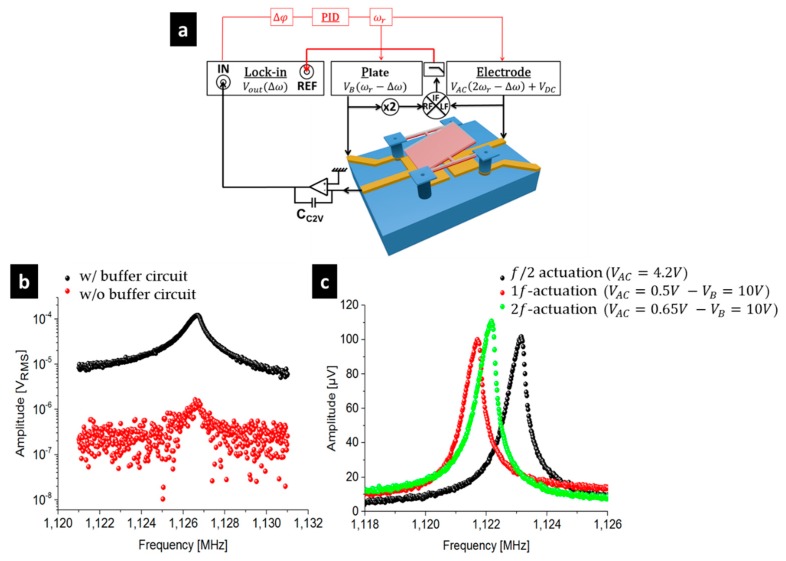
Enhancement provided by the buffer circuit: (**a**) Synopsis showing the set-up to characterize the pixels in a down-mixed readout scheme (in open loop or in a closed-loop: red part); (**b**) Comparison of the output signal between a semitone down-mixing approach with the buffer circuit and without the buffer circuit (the polarization voltages are explained in Table 2); (**c**) Typical output signals for the three down-mixing approaches with the buffer circuit. At resonance, the capacitance variation is close to 10 aF. The SNR is larger than 40 dB for the three cases.

$f_{CO}$ is the high-pass cut-off frequency of the amplifier, and $f_{c}=\frac{1}{2\pi R_{FB}C_{C2V}}$ the low-pass frequency at −3 dB of the RC filter. The improvement that was brought by the buffer circuit is illustrated in Figure 6. With our buffer, the SBR was kept quite constant, while the SNR strongly improved, growing up to 42 dB (to be compared with 20 dB without the buffer).

The output voltages at resonance were high and clean enough to embed the electromechanical pixels into a closed loop. At this stage, the pixels of the array of 96 × 96 pixels (see Figure 3a,b) have been tested using an external closed-loop based on a down-mixed phase locked loop (PLL) scheme—only 96 × 96 pixels among the 666 × 520 pixels of the complete array (see Figure 3) could be effectively electrically controlled for use in this first proof of concept. The latter is shown in red in Figure 6a. To do so, the output phase signal Δφ (demodulated at Δf) was the input signal of a digital proportional–integral–derivative controller (PID). The output $f_{r}$ corresponded to the frequency applied to the pixel. The PID parameters were set according to the Ziegler-Nichols method [46], hence modifying the bias voltage $V_{B}$ applied on the pixel. This voltage enabled the control of the effective stiffness [47].

The next section will provide typical electromechanical results and the thermal sensitivity of our electromechanical system. The noise sources of such a system are presented. Based on these measurements, new readout schemes of large pixel arrays are suggested, to achieve a compact CMOS circuit beneath the imager.

## 3. Results

### 3.1. Electromechanical Characterizations

**Figure 7:**
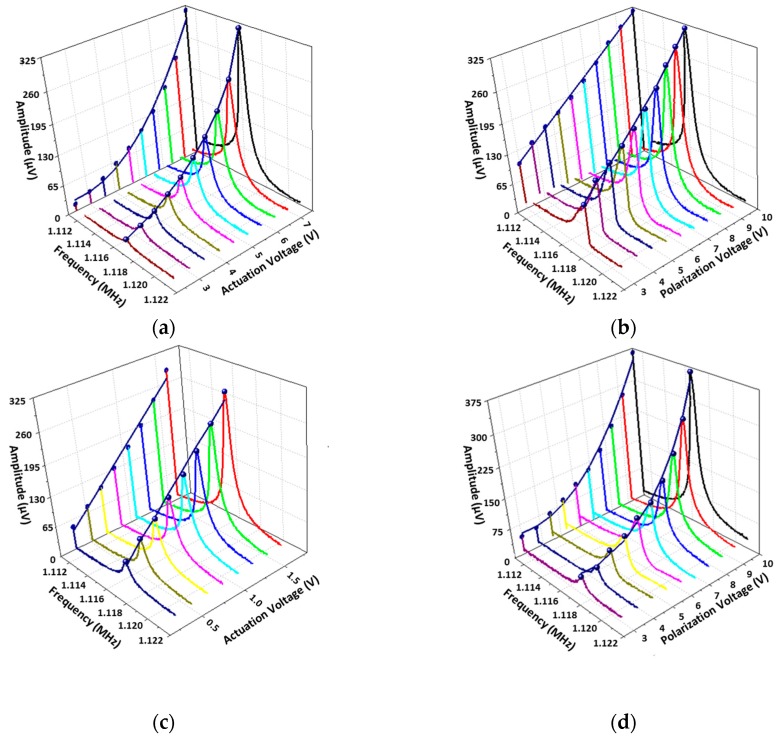
Electromechanical response when the frequency is swept around the resonance for different actuation schemes: (**a**) Amplitude versus f and $V_{AC}\;$ for a semitone actuation $\left(V_{B}=10\;V\right)$; (**b**) Amplitude versus f and $V_{B}\;$ for a semitone actuation $\left(V_{AC}=6.5\;V\right)$; (**c**) Amplitude versus f and $V_{AC}\;$ for a 2*f*-actuation $\left(V_{B}\;=10\;V\right)$; (**d**) Amplitude versus f and $V_{B}\;$ for a 2*f*-actuation $\left(V_{AC}=1.8\;V\right)$.

First, the electromechanical responses of the pixels of the 96 × 96 array were measured in an open loop. The variations of the resonance amplitude as a function of the voltages $V_{B0}$ and $V_{AC0}$ were verified for the *f*/2 and 2*f* actuation schemes, up to the onset of non-linearity (i.e., $\theta_{c}\sim 17{}^{\circ}$). Figure 7a,b correspond to a *f*/2-actuation showing, as expected, a quadratic variation of the output voltage at resonance with $V_{AC0}$ and a linear variation with $V_{B0}$ respectively. Figure 7c,d correspond to the 2*f*-actuation showing a linear variation of the output voltage with $V_{AC0}$ and a quadratic variation with $V_{B0}$, which also expected.

The main electromechanical features for the torsional mode ($f_{0},\;Q$ and $V_{out}$, the maximum output voltage corresponding to the onset of nonlinearity of the deflection angle $\theta_{c}$) were measured on every pixel of a 96 × 96 array with the 2f-actuation scheme:

- $f_{0}=\left[1.05–1.2\;\mathrm{MHz}\right]$;
- Q=[1600–2500];
- $V_{out}=\left[100–350\;µV\right]$

The range of resonance frequencies was coherent, with a fabrication process dispersion of 10% on the torsional rod width (length and thickness variations were negligible). The variations on the quality factor and the maximum voltage were rather more sensitive to the mechanical anchoring of the insulation legs, and the over-etching effect between the edge and the center of the array explained this difference. Spurious flexural motions of the insulation legs (perpendicular to the rods) may have impacted the torsional vibration. At first insight, it increased the damping rate and lowered the effective torsional stiffness. In a more complete approach, the real anchoring features were included into a nonlinear model that was previously presented [47]. In particular, the anchoring was modelled with a stiff spring along the *z*-axis. We demonstrated that the onset of nonlinearity can be decreased depending on many other factors (the electrostatic torques…), hence modifying the detector sensitivity. In the worst cases, the thermal sensitivity may be decreased by 10%, compared to the value that is expected with a perfect anchoring.

The frequency dispersion does not impact the closed-loop performance, and it has a tiny impact on the thermal response (see Equation (1)). However, the quality factor and the dynamic range had a larger impact on the noise floor level, as we will see later in Section 3.2. This means that the pixels on the edges of the future imager will be slightly less sensitive compared to the others. To go further on this topic, the next section will address the noise of the readout chain and the thermal performance of such pixels.

### 3.2. Thermal Characterizations

Let’s go back to Equation (1), which gives the thermal response to an incident IR-radiation. The frequency shift is proportional to the temperature coefficient of frequency $\alpha_{T}$, and inversely proportional to the thermal conductance G of the material stack of the rods and the insulation legs.

#### 3.2.1. TCF & *G*

Using the closed loop (Figure 5a), we implemented systematic TCF measurements on typical devices (Figure 3c and Figure 4a–c). In order to carry out a large number of measurements within a reasonable time, the devices were tested on an automatic probe-station that was dedicated to 200 mm wafers. The latter was heated with a hotplate to have a temperature variation between 0° and 20° above the ambient temperature. Beyond this limit, the closed-loop did not track the frequency shift anymore. The measurements were performed with a coupled Peltier-Pt sensor controlled by a Proportional Integral Derivative controller (PID) to obtain the chamber temperature (down to 0.1 °C-accuracy). The statistics are summarized in Table 3.

The TCF of a typical pixel (55.4 ppm/°C) was in good agreement with the theoretical value of 48 ppm found by finite element method simulation (FEM) on our stack. In the case of pixels with thin nano-rods, the axial internal stress was higher and its variation with temperature reinforced the TCF (same sign of variation). In the meantime, the thermal insulation was quite well enhanced. Thus, a global improvement of the thermal response should be expected with this kind of pixel.

#### 3.2.2. Thermal Response

**Figure 8:**
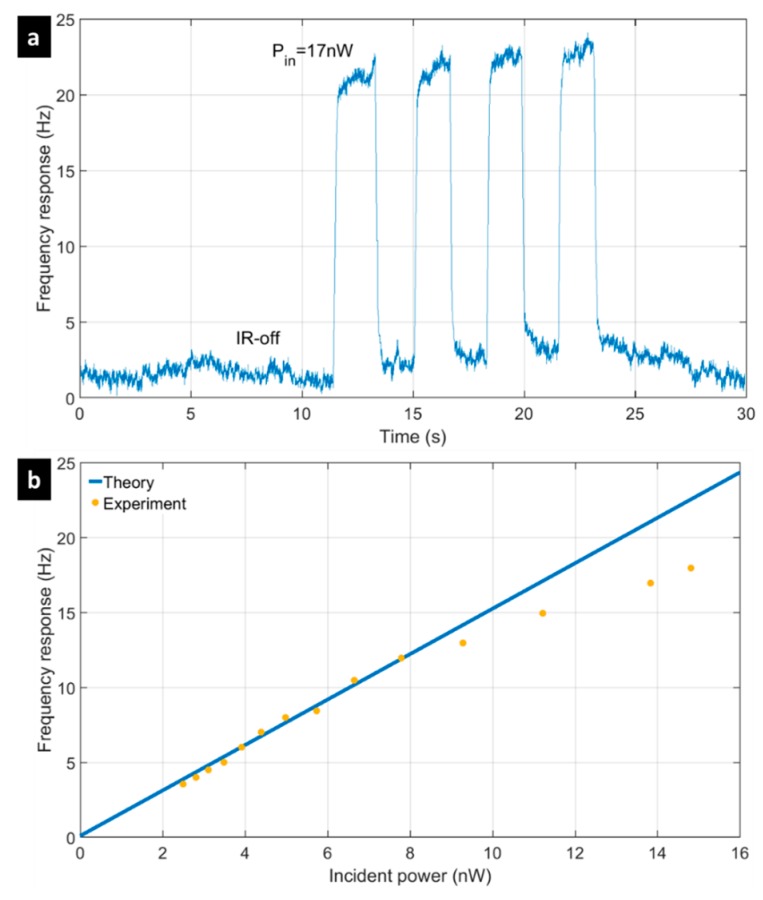
Measurement of the thermal response: (**a**) Resonance frequency shift induced by incident IR pulses (peaks of 17 nW); (**b**) Frequency shift when the incident power is varied from 2 to 16 nW.

The thermal response was measured and compared with the theoretical values computed by FEM. Measurements were performed with the readout chain shown in Figure 5a in closed-loop. The device under test (a pixel array) was placed into a vacuum chamber, and a blackbody source (RCN 1200 from HGH Infrared Systems set at 1200 °C) was positioned in front of it. An 8–12 µm-filter was put between our chamber and this source, to control the incident power. The optical bench was aligned thanks to a visible laser. The optical set-up was calibrated using a Fourier transform infrared instrument (FTIR). In particular, the spectral response of the filter according to the spectral luminance of a perfect blackbody at 1200 °C was measured. A photometric computation (knowing the optical apertures and the relative distances between the optical blocks) was used to determine the incident optical power. We considered that the source is a Lambertian black body with a monochromatic luminance, described by Planck’s law. The aperture of the source and the chamber window were close enough to neglect the atmosphere absorption in the estimation of incident power (d=2.5 cm). The frequency response of our typical pixel-to-IR incident pulses (17 nW peaks) is presented in Figure 8a). Thermal responses up to *R_f_* = 1050 W^−1^ were extracted with the best devices ($f_{0}=1.15\;\mathrm{MHz})$. Assuming a fill factor *β* = 0.8, an efficiency *η* = 0.8 in the 8–12 µm window, and considering the measured TCF, *α_T_* = −76 ppm/°C, a theoretical thermal response *R_f_* = 950 W^−1^ was expected, which was very close to the observed sensitivities. In a second experiment, the incident IR flux on a pixel was changed by varying the distance between the window and the IR-source. The frequency shift was then the measured for optical powers varying from 2.5 to 16 nW. The experimental results and their linear fit are presented in Figure 8b. A thermal response of *R_f_* =1350 W^−1^ was extracted from the slope, considering the resonance frequency mentioned above. Above 8 nW, the relationship between the IR-flux and the thermal frequency shift was no more linear. To increase the incident power, the source was moved closer to the window, which caused it to heat up. This effect lowered its transmittance, resulting in an incorrect estimation of the thermal response (namely, *R_f_* =1050 W^−1^).

Similar experimental thermal responses were extracted on other pixels of a same array. From one array to another, experimental *R_f_* varied from 700 to 1350 W^−1^, showing some dispersion attributed to the fabrication process.

#### 3.2.3. Response Time

**Figure 9:**
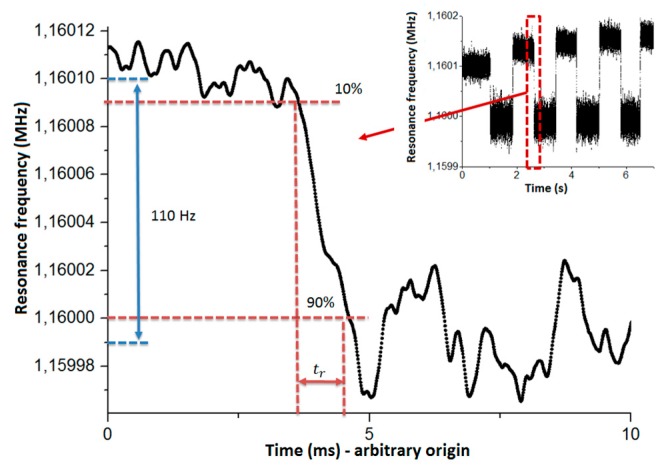
Resonance frequency according to the acquisition time. A 1 mW red laser is focalized onto the pixel under test (typical Figure 3c). Insert: Full data from our measurement of response time. The average frequency jump is estimated as 110 Hz. Then, the response time is extracted from one event fall time (red).

The response time was measured with a helium neon laser (633 nm) from a commercial Polytec vibrometer (see Figure 9). Since the needed integration time was too short (180 μs) compared to the PLL time constant, this experiment was performed in an open loop scheme (see Figure 6a). We verified that the response time was not limited by the electrical low-pass filter from our measurement set-up, by setting *τ* = 50 µs. The optical power was set to remain in the linear dynamic range (±13.5° for the typical design). The 10–90% method was used to extract the fall time $t_{r}$, and the response time of the first order low-pass filter $\tau \;\left(\tau \;=\;t_{r}/\ln\left(9\right)\right)$. By doing so, we extracted a response time of 430 µs, which was close to the theoretical value computed with the thermal equations (500 µs). The resonant electromechanical pixels could follow quite quick events in the scene. They had a faster response than the current resistive pixels.

### 3.3. Noises and Temperature Sensitivity

The performances of the electromechanical pixels were estimated through the noise equivalent power (NEP) or the NETD. The NEP is defined as the incident power on the sensor surface with a SNR of 1. This corresponds to the minimum measurable frequency shift:

(7) $$\mathrm{NEP}=\frac{1}{R_{f}}\frac{<\delta f^{2}{>}^{1/2}}{f_{0}}=\frac{\sigma_{y}}{R_{f}}$$

$<\delta f^{2}{>}^{1/2}$ is the rms frequency fluctuation for a given bandwidth, and $\sigma_{y}$ is the quadratic deviation of the instantaneous relative frequency.

Current “column” or “rolling-shutter” readout schemes should be implemented with our resonator array. The column readout, which is the current approach with CMOS circuit, requires a 60 Hz frame rate, which sets a pixel integration bandwidth at 7 kHz for 190 pixels per column, for instance [48]. However, this readout scheme may induce a lag effect leading to an image distortion when the scene moves faster than the frame rate. A single-pixel readout is a solution to remove this effect. In this case, the integration time corresponded to the full frame rate, i.e., 50 Hz, thereby increasing the SNR. As the capacitive detection did not suffer from self-heating issue, it was possible to use a longer integration time without material degradation, even for small pitches below 12 µm. This is why the NEP of our sensor was estimated for three noise bandwidths, $f_{BW}=\;$7 kHz, 50 Hz, and 10 Hz.

Let’s get back to a few computational and theoretical considerations to understand and estimate the different noise sources contributing to $\sigma_{y}$. The overall $\sigma_{y}$ is the quadratic sum of these noise contributions that are considered as uncorrelated: $\sigma_{y}=\sqrt{\sum_{i}\sigma_{yi}^{2}}$, where *i* corresponds to: (1) the thermomechanical noise, which is due to the coupling with an ambient thermal bath; (2) the readout electronics’ noise, and (3) the phonon noise.

$\sigma_{y}$, whose main origin is the thermomechanical noise, is inversely proportional to the SNR [28,49]:

(8) $$\sigma_{y}^{Th}=\frac{1}{2Q}\frac{\sqrt{\langle \theta_{n}^{2}\rangle }}{\theta_{c}}=\frac{1}{2Q}\frac{1}{\mathrm{SNR}}$$

where $\langle \theta_{n}^{2}\rangle$ is the thermomechanical deflection noise. The above expression shows that the linear range and the quality factor must be as high as possible to obtain a stable oscillator. This noise can be estimated through the Parseval-Plancherel theorem: $\langle \theta_{n}^{2}\rangle =\sqrt{\int_{f_{0}\;-\;\frac{\Delta f}{2}}^{f_{0\;}\;+\;\;\frac{\Delta f}{2}}S_{\theta }\left(f\right)df}$. Using the dissipation-fluctuation theorem, the power spectral density of the thermochemical noise can be written as [50]: $S_{\theta }\left(f\right)=\left(4\pi k_{B}T/Q\right)\kappa f_{0}^{3}/{\left((f_{0}^{2}\;-\;f^{2}\right)}^{2}\;+\;\left(ff_{0}/Q\right)²)$, with: $k_{B}$ as the Boltzmann constant, and *T* as the ambient temperature.

When $f\;\ll \;f_{0}/Q$ (i.e., when the readout bandwidth is smaller than the mechanical response time; in other words, for $f_{BW}=50\;\mathrm{Hz}$), this expression is simplified: $\langle \theta_{n}^{2}\rangle =\frac{2k_{B}TQ}{\pi \kappa f_{0}}\sqrt{f_{BW}}$. Interestingly, at a fast integration time (i.e., $f_{BW}=7\;\mathrm{kHz}$), $\langle \theta_{n}^{2}\rangle =k_{B}T/\kappa$, which corresponds to the equipartition energy theorem.

Similarly, $\sigma_{y}$, whose origin is the readout electronics noise, is expressed as:

(9) $$\;\sigma_{y}^{elec}=\frac{1}{2Q}\frac{\sqrt{v_{n}^{2}}}{V_{0ut}}$$

where $\langle v_{n}^{2}\rangle$ is the readout electronics noise generated by the buffer circuit (see Figure 5).

The fundamental source of noise for a thermal conductance that is higher than the radiation conductance [47] should be the phonon noise that results from the random exchange of heat between the sensor and the thermal bath through the mechanical anchors. At thermodynamic equilibrium, the temperature fluctuations due to this fundamental phenomenon can be written as:

(10) $$\;\sigma_{y}^{phonon}=\alpha^{2}<\bar{\Delta T}{>}^{2}=\{\begin{array}{c} \frac{4\alpha^{2}k_{B}T^{2}}{G_{th}}\Delta f\;if\;f_{BW}\;<\;f_{th}\;\\ \frac{\alpha^{2}k_{B}T^{2}}{C_{th}}if\;f_{BW}\;>\;f_{th} \end{array}$$

with $f_{th}=1/4\tau_{th}$ as the thermal cut-off frequency.

The direct phase or frequency fluctuations can be expressed according to a sum of frequency sources with the spectral power density: $S_{y}\left(f\right)=Kf^{\alpha }$ (−4 < α < 2).

The orders of magnitudes of the noises and their consequence on the frequency stability and NEP are summarized in Table 4 below, for the two considered bandwidths. We noticed that the readout electronics noise drastically degraded the performance of such a system. In comparison, the NEP of a classical resistive 12 µm-pitch pixel was around 30 pW. This performance could be reached in principle if the electronics noise was minimized. We also mentioned the NEP for another integration time ($f_{BW}=1\;\mathrm{Hz}$) that was rather used for gas or mass measurements. If a new readout strategy could be defined with 1s-integration time, the performance would even be better than the current bolometers.

Experimental measurements of the frequency stability were achieved to verify our assumption and to think about a specific readout strategy for our electromechanical array. To this end, the frequency stability was measured in closed-loop by estimating the Allan deviation $\sigma_{A}$ of the output signal according to the integration time τ. This deviation is a typical tool for the estimation of the stability of oscillators [51]; in particular, to characterize their long-term drift. However, the main types of noises can easily be observed with such a mathematical tool. In particular, a fluctuation of the ambient temperature surrounding the sensor will have an impact that is directly observed on the long term noise. This effect is directly taken into account in the Allan deviation measurements. Besides this point, the sensor has to include blind pixels that provide information on the ambient temperature. The frequency power spectral density was also measured on the same typical pixel. 

**Figure 10:**
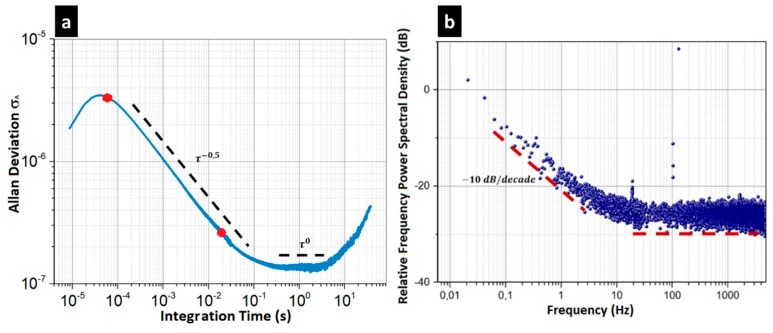
Noise characterization achieved on the typical electromechanical pixel—the amplitude at resonance is set at 320 µV: (**a**) Allan deviation measurement; the red hexagon indicates the frequency deviation for $f_{BW}=7\;\mathrm{kHz}$ ($\sigma_{A}=3.5\times 10^{-6}$ ) and the red disk is the one for $f_{BW}=50\;\mathrm{Hz}$ ($\sigma_{A}=3\times 10^{-7})$; a plateau is reached between 50 and 200 ms integration time ($\sigma_{A}=1.5\times 10^{-7}$ ); beyond 200 ms, a strong drift effect can be observed; (**b**) spectral power density measurement achieved on the same device. The Allan deviation has a $\tau^{-\frac{1}{2}}\;$ stop at a short integration time corresponding to the signature of a White amplitude noise. Beyond 50 ms integration time, the Allan deviation presents a plateau, which shows a 1/*f* frequency noise. These two noises can also be distinguished on the power spectral density: the slope of −10 dB/decade corresponds to this plateau.

The Allan deviations measured in the closed loop are presented in Figure 10a for the typical pixel. Between 70 µs and 50 ms $\sigma_{A}$ dropped with a $\tau^{1/2}$ slope, showing that white noise was the main contributor in this interval. As shown in Table 4, this trend was mainly attributed to our readout electronics, whose amplitude level was measured at around $40\;\mathrm{nv}/\sqrt{\mathrm{Hz}}$. A plateau at $1.5\times 10^{-7}$, appeared between 50 and 200 ms. This 1/*f*-noise was well above the noise floor that was normally set by the thermomechanical white noise and phonon noises (close to few $10^{-8}$ for the two noise sources). Supplementary experiments were achieved to try to understand the origin of this 1/f-noise. In particular, the Allan deviation was measured for different actuation voltages $V_{AC}$ to increase the maximum output voltage and to improve the SNR. We demonstrated that the plateau is independent of the SNR. We believe that this noise floor is fully inherent in pure frequency fluctuations, whose origin is not clearly identified. Similar noise signatures have been reported as an anomalous phase noise (APN) for flexural nanoresonators [52]. This fundamental noise would only be relevant for small vibrating bodies, which is the case for the nanorods used in the pixel. In the first conclusion, the stability limit of our torsional resonators was set by the APN, and had to be considered as the fundamental limit of our resonant sensors. Even for a 1 s integration time, the NEP would be stuck at around 100 pW. In the discussion section below, we try to figure out this issue.

## 4. Discussion

First, for the sake of clarity, the NETD, which is basically the lowest temperature variation that is detectable on the scene, is computed for our devices. NETD is directly proportional to the NEP and thus proportional to the frequency stability:

(11) $$\mathrm{NETD}=\frac{4F^{2}}{\pi A_{p}\Phi_{\lambda_{1}\to \lambda_{2}}{\left(\frac{\Delta L}{\Delta T}\right)}_{{300K}_{\lambda_{1}\to \lambda_{2}}}}\times \frac{\sigma_{y}}{R_{f}}\;$$

where F is the optical aperture (usually F=1), $A_{p}$ is the pixel area, Φ and (ΔL/ΔT) are the optical transmission and the luminance variation with a scene temperature of around 300 K, are both evaluated in the $\left[\lambda_{1};\lambda_{2}\right]$. range. In the 8–14 µm range, Φ is usually close to one, and (ΔL/ΔT) is evaluated as 0.84 W/m^2^/sr/K [53].

NETD was computed from the experimental Allan deviations and thermal responses with Equation (11). As a figure of merit, $FOM=NETD\times \tau_{th}$, is usually introduced to evaluate in a glance the quality of a microbolometer technology [54]. Actually this *FOM* avoids any dependence of the sensor performance to the thermal conductance. These two parameters are shown in Table 5 for our electromechanical components and a current resistive pixel is used as a reference.

A quick insight in Table 5 shows that the NETD of our components cannot compete with the temperature performance of a resistive pixel. Experimental NEP at 50 Hz and 7 kHz bandwidths are close to values obtained through Equations (7), (9), and (11) considering $R_{f}=1050\;/W$ and the experimental value of the electronics noise, $40\;\mathrm{nV}/\sqrt{\mathrm{Hz}}$. The NETD of 2 K at 50 Hz and 1.5 K at 100 ms—with a sub-millisecond response time for 8–12 µm incident radiation—were extracted. The usual integration times correspond to the white noise region of our resonator (see Figure 10a) and the NEP can be expressed in terms of a single power density of $30\;\mathrm{pW}/\sqrt{\mathrm{Hz}}$. At a long integration time (at 10 Hz), the NETD is set by the APN. In principle, the improvement of the electronics or the thermomechanical noises will not positively influence this limit for long integration times. This analysis tells us that the readout scheme per column at 7 kHz currently used for the resistive bolometer is not suitable for our approach.

From these first conclusions, and if we look at the Equation (11), a few clear avenues of improvement can be proposed:

- Frequency stability and matrix readout strategy: A 50 Hz integration bandwidth requires an improvement of the noise amplitude of our buffer electronics close to the pixel. Lower amplitude noise levels can be reached by using self-oscillating electronics requiring only a few transistors, unlike PLL circuits. Moreover, our electronics was realized close to the pixel but this was not done through an application-specific integrated circuit (ASIC) fabricated underneath the electromechanical pixels. The low-temperature fabrication process presented above has already been used to manufacture resistive bolometer imagers on top of CMOS circuits (ROIC) by post-processing [37,55], and it should be straightforward to reuse this approach in our case. As mentioned in the introductive section of the paper, a co-integration of the readout electronics at the pixel level will reduce the parasitic capacitance down to a few fF, and will decrease the electrical noise down to a theoretical level of $10\;\mathrm{nV}/\sqrt{\mathrm{Hz}}$, or even $5\;\mathrm{nV}/\sqrt{\mathrm{Hz}}$. This approach makes the down-mixing detection scheme unnecessary, leading to a much simpler measurement chain than the strategy presented here. $\sigma_{y}$ will be decreased by a factor of 8 with a self-oscillating IC (a gain of a factor 4 on the absolute noise, and a gain of a factor 2 on the output voltage with a the direct detection (see Equation (9))). Thus, the electronics noise will become lower than the fundamental APN ($\sigma_{y}=1.5\times 10^{-7}$) for a 700 Hz integration bandwidth. This conclusion leads us to suggest a new readout scheme consisting of reading 700/50 = 14 pixels during a 50 Hz frame rate, which allows for a larger area for the co-integrated readout. These two straightforward improvements allow us to obtain a *FOM* that is close to 0.75 for a $f_{BW}=50\;\mathrm{Hz}$ (global shutter approach), which is an encouraging element.
- Thermal response: At the end, the noise floor level will be set by the APN, whatever the electronics and the readout strategy. An improvement of the signal through the thermal insulation 1/G is much trickier in our case. Indeed, this would require long and thin rods/insulations legs, and this would lower the onset of nonlinearity of $\theta_{c}$ (see Equation (8)), leading to a degradation of the SNRs and therefore the frequency stability $\sigma_{y}$.

A simple look at Equation (11) demonstrates that the thermal response $R_{f}$ has to be increased through the TCF (the temperature coefficient of resistance of a resistive pixel is around 2% whilst the TCF is around 50 ppm). To date, we did not observe a major difference between the experimental TCF values ranging from −35 ppm/°C to −100 ppm/°C. Unfortunately, the highest TCF occurs with soft devices, which were not suitable for IR sensing, as explained above.

**Figure 11:**
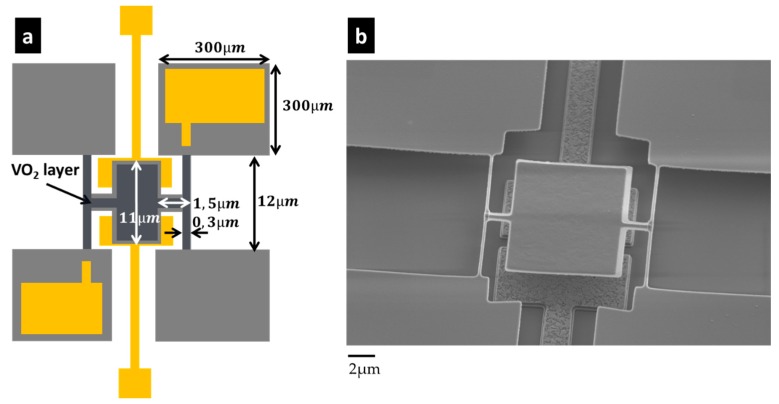
Torsional resonator design: (**a**) Schematics of the design; (**b**) SEM image of a typical pixel with a VO_2_ layer on top of both the plate and torsional rods, plus partially on the insulation legs.

We will thus focus the discussion on increasing the TCF of our devices. Some interesting works have already shown a TCF up to 1000 ppm/°C, thereby improving the TCF by a factor of 20 [56,57]. In particular, the first-order phase transition of diverse materials has been used to obtain Young’s modulus, which are highly sensitive to temperature [58,59]. Following this line, we are manufacturing similar 12 µm-pitch electromechanical pixels, including VO_2_ materials, on top of our pixel. This material was deposited in its amorphous state by reactive deposition (Ion Beam Deposition) and annealed at 400 °C to obtain the crystalline state. The process temperature is kept low enough to be used in a post-process of a CMOS circuit. The Raman characterizations were done to verify the crystallization obtained with this method. The resonators were designed to keep the mechanical features of our current typical pixel (Figure 3c). Nano-indentation measurements were performed on a full layer to extract the Young’s modulus of our VO_2_ layer (177 GPa for the crystalline state and 80 GPa for the amorphous state). A thickness of 80 nm was then chosen with 1.5 µm long and 300 nm wide torsional rods. In an initial version, both the rods and the plate are covered with the VO_2_ layer, and in a second version, the VO_2_ layer is only left on the rods. An example of the fabricated devices is shown in Figure 11. The TCF measurements and then the frequency stability are ongoing. We expect an improvement of one order of magnitude in the thermal response (the mechanical features and the thermal insulation being kept constant compared to the standard pixel).

## 5. Conclusions

Our electro-optical measurements show that our current electromechanical resonant pixels cannot compete with the best 12 µm pitch resistive bolometer in terms of NETD. Three major straightforward improvements can be done: (1) the buffer and the electronics readout, including the addressing circuit, has to be included into a ROIC directly beneath the imager as the bolometer. Doing so, the electronics noise and the parasitic capacitance will be negligible, regarding the other sources of noises. The noise level will be set by the APN, which is the fundamental limit of such an approach. This limitation can be overcome by increasing it by a factor of 10- or 100-fold greater than the frequency response. We showed in this paper one of the more promising ways to reach this goal, by integrating phase transition materials on top of the rods. The first realizations demonstrated that we were able to reproduce the same device without thermal features degradations. The optimizations of the pixel are on-going. With both the improvements of the frequency response of a factor of 10, and a pixelwise readout, or at least a readout of 14 in the same frame rate, the NEP would be lower than 20 pW (i.e., NETD<180 mK). The *FOM* would drop to 0.09, and this value is comparable with the current technology (*FOM* = 0.05). Based on this projection, we believe that the uncooled IR sensors based on the nanomechanical resonators will experience a new interest for small pitches below 12 µm. 

## Figures and Tables

**Table 1:** Key parameters presented in the Equations (1)–(5) for our device compared with an advanced resistive bolometer and microelectromechanical systems (MEMS) bolometer: temperature sensitivity corresponds to $\frac{1}{f}\;\times \;\partial f⁄\partial T$ for a resonant thermal sensor and $\frac{1}{R}\;\times \;\partial R⁄\partial T$ for a resistive one.

Electromechanical & Thermal Features	This Work (Figure 3c)	Bolometer [32]	Resonant MEMS [9]
Maximal Angle $\;\theta_{max}$ (°)	21	N.A	-
Inertial Moment J (kg.m^2^)	$3.9\times 10^{-25}$	N.A	$1.5\times 10^{-27}$
Torsional stiffness κ (N.m)	$1.8\times 10^{-11}$	N.A	$6.8\times 10^{-13}$
Resonant Frequency (MHz)	1.1	N.A	-
Onset of Nonlinearity $\;\;\theta_{c}$(°) (This value is computed by solving a nonlinear dynamic equation [44].)	13.5	N.A	-
Quality Factor Q	1800	N.A	1555
Capacitance at Rest $C_{0}$ (fF)	0.185	N.A	N.A
Pitch (µm)	12	12	5
Thermal Conductance G (W/K)	$5\times 10^{-8}$	$5\times 10^{-9}$	$1.5\times 10^{-8}$
Thermal Capacity C (J/K)	$26\times 10^{-12}$	$80\times 10^{-12}$	$3\times 10^{-12}$
Thermal Constant $\tau_{th}$ (ms)	0.5	16	0.2
Temperature Sensitivity (/°C)	0.01%	3.6%	0.0092%

**Table 2:** Preliminary measurement of the signal-to-background ratio (SBR) for different transduction strategies (direct semitone, direct 1f, differential and down-mixed); $V_{B0}=10\;V$ , $V_{DC}=10\;V$ and $f_{0}=1\;\mathrm{MHz},\;\Delta f=10\;\mathrm{kHz}$; $V_{AC0}=4.2\;V$ for semitone actuation and $V_{AC0}=0.5\;V$ for 1*f* and 2*f* actuations.

Transduction Method	Voltages		SBR (dB)
-	$V_{AC}$	$V_{B}$	-
1f-actuation	$V_{AC0}\cos\left(2\pi ft\right)$	$V_{B0}$	−33
f/2-actuation	$V_{AC0}\cos\left(\frac{2\pi ft}{2}\right)$	$V_{B0}$	−13
f/2-actuation/differential mode	$V_{AC0}\cos\left(\frac{2\pi ft}{2}\right)$	$V_{B0}$	2
f/2-actuation/down-mixing mode	$V_{AC0}\cos\left(\frac{2\pi ft}{2}\right)$	$V_{B0}\cos\left(2\pi ft+\Delta f\right)$	22
f-actuation/down-mixing mode	$V_{AC0}\cos\left(2\pi ft\right)\;+\;V_{DC}$	$V_{B0}\cos\left(2\pi ft+\Delta f\right)$	20
2f-actuation/down-mixing mode	$V_{AC0}\cos\left(4\pi ft+\Delta f\right)$	$V_{B0}\cos\left(2\pi ft+\Delta f\right)$	22

**Table 3:** Temperature coefficient of frequency (TCF) measured on different types of pixels: mean and standard deviation per wafer; thermal conductance of rods & legs G (computed from material properties and geometry measured by SEM).

Pixel Types	$\langle \alpha_{T}\rangle \;(\mathrm{ppm}/{}^{\circ}C)$	$\sigma_{\alpha_{T}}$	G (W/K)	$\langle \alpha_{T}\rangle /G$
Typical (Figure 3c)	55.4	14.6	$5\;\times 10^{-8}$	$1.11\times 10^{9}$
Butterfly (Figure 4a)	45.2	3.6	$3.10\times 10^{-8}$	$1.46\times 10^{9}$
Typical with Thin Nano-Rod (Figure 4c)	86.2	16.4	$1.8\times 10^{-8}$	$4.79\times 10^{9}$

**Table 4:** Theoretical frequency stability and NEP for the nominal pixel with our electronics and 2*f*-down-mixing readout scheme: $R_{f}=1050\;/W$, $\theta_{C}=13.5{}^{\circ}$, $V_{n}=10\;\mathrm{nV}/\sqrt{\mathrm{Hz}}$, Q=2500 and $V_{out}=320\;µV$.

Noise Sources	$f_{BW}\;=\;50\;\mathrm{Hz}$			$f_{BW}\;=\;7\;\mathrm{kHz}$			$f_{BW}\;=\;1\;\mathrm{Hz}$
	$X_{n}$	$\sigma_{y}$	NEP	$X_{n}$	$\sigma_{y}$	NEP	NEP
Thermodynamic	$3.6\times 10^{-6}\;\mathrm{rad}$	$6.3\times 10^{-9}$	6 pW	$1.2\times 10^{-5}$ rad	$2.6\times 10^{-8}$	25 pW	0.85 pW
Electronics	70.7 nV	$8.9\times 10^{-8}$	85 pW	836.7 nV	$1\times 10^{-6}$	1000 pW	12 pW
Phonon	-	$5.8\times 10^{-9}$	5.5 pW		$1.8\times 10^{-9}$	17 pW	0.8 pW

**Table 5:** Comparison between our pixels and a classical resistive bolometer for three integration bandwidths—all electromechanical devices are set at their onset of nonlinearity.

Pixel	$\tau_{th}$ (ms)	$R_{f}$ $\left(/W_{inc}\right)$	$\mathrm{NEP}\;f_{BW}=10\;\mathrm{Hz}\;(\mathrm{nW})$	$\mathrm{NEP}\;f_{BW}=50\;\mathrm{Hz}\;(\mathrm{nW})$	$\mathrm{NEP}\;f_{BW}=7\;\mathrm{kHz}\;(\mathrm{nW})$	NETD $(K)\;(\mathrm{FOM}—K\cdot \mathrm{ms})\;f_{BW}=10\;\mathrm{Hz}$	NETD $(K)\;(\mathrm{FOM}—K\cdot \mathrm{ms})\;f_{BW}=50\;\mathrm{Hz}$
Typical	0.5	1050	0.14	0.19	2.4	1.5 (0.75)	2 (1)
Butterfly	0.8	1011	0.47	1.1	12	4.9 (3.96)	11.6 (9.28)
Thin Rod	2.8	3555	1.3	3	35	13.7 (38.3)	-
Resistive Pixel [1]	16	-	-	0.05	-	-	0.05 (4)
